# Simultaneous Multicolor Multifocal Scanning Microscopy

**DOI:** 10.1021/acsphotonics.3c00205

**Published:** 2023-07-24

**Authors:** Kyungduck Yoon, Keyi Han, Kidan Tadesse, Biagio Mandracchia, Shu Jia

**Affiliations:** † Wallace H. Coulter Department of Biomedical Engineering, Georgia Institute of Technology and Emory University, Atlanta, Georgia 30332, United States; ‡ Parker H. Petit Institute for Bioengineering and Biosciences, Georgia Institute of Technology, Atlanta, Georgia 30332, United States; § George W. Woodruff School of Mechanical Engineering, Georgia Institute of Technology, Atlanta, Georgia 30332, United States

**Keywords:** MSM, super-resolution systems, optical sectioning, super-resolution imaging, image-scanning microscopy, cell biology

## Abstract

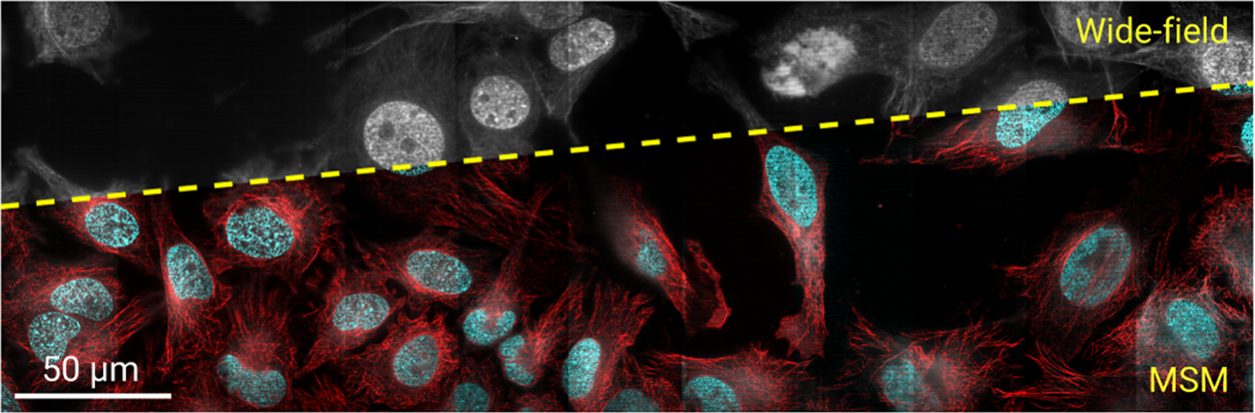

Super-resolution fluorescence microscopy has revolutionized cell biology over the past decade, enabling the visualization of subcellular complexity with unparalleled clarity and detail. However, the rapid development of image-scanning-based super-resolution systems still restrains convenient access to commonly used instruments such as epi-fluorescence microscopes. Here, we present multifocal scanning microscopy (MSM) for super-resolution imaging with simultaneous multicolor acquisition and minimal instrumental complexity. MSM implements a stationary, interposed multifocal multicolor excitation by exploiting the motion of the specimens, realizing super-resolution microscopy through a general epi-fluorescence platform without compromising the image-scanning mechanism or inducing complex instrument alignment. The system is demonstrated with various phantom and biological specimens, and the results present effective resolution doubling, optical sectioning, and contrast enhancement. We anticipate MSM, as a highly accessible and compatible super-resolution technique, to offer a promising methodological pathway for broad cell biological discoveries.

## Introduction

The advancement of super-resolution imaging techniques over the past decades has overcome the physical barrier of conventional optical microscopy, allowing for sub-diffraction-limited visualization of subcellular organization with unprecedented details.^1−3^ Among these developments, structured illumination microscopy (SIM) techniques achieve optical super-resolution by recovering structural details that contain high-spatial frequencies through spatial-frequency mixing.^4,5^ Today, SIM has evolved as one of the most widely implemented techniques, featuring effective resolution improvement, good acquisition speed, and high compatibility with standard sample protocols.^6−9^

In particular, the recent advance in image-scanning microscopy (ISM), a confocal form of SIM, extends the super-resolution performance of traditional interference-based SIM.^10,11^ In principle, ISM utilizes each pixel in a detector array as a confined pinhole for scanning diffraction-limited laser focus or multiple foci,^12^ thereby offering enhanced optical sectioning, uncompromised signal-to-noise ratio (SNR), and rapid acquisition.^10,11,13−17^ However, the broader applicability of ISM remains limited due to existing optical configurations. For instance, current ISM systems are still complicated by the scanning implementations, such as confocal spinning disks, galvanometric mirrors, or digital micromirror devices, which restrains convenient access to commonly used instruments such as epi-fluorescence microscopes. In addition, to acquire multiple cellular entities, existent strategies accommodate multicolor imaging by splitting the imaging sensor,^18^ incorporating multiple cameras,^19^ or sequential acquisition of spectral channels,^13,16^ which may inevitably compromise the imaging ability (e.g., the field of view, speed) and instrument simplicity.

To address these problems, we present multifocal scanning microscopy (MSM), an ISM system allowing super-resolution imaging with simultaneous multicolor acquisition and minimal instrumental complexity. In particular, unlike existing spot-scanning schemes, MSM implements a stationary multifoci configuration by taking advantage of the motion of the specimens, realizing super-resolution microscopy through a general epi-fluorescence platform. Furthermore, MSM forms a multifocal excitation pattern that consists of equally sized and evenly distributed multicolor arrays, which facilitate the simultaneous acquisition of multiple cellular components without compromising the image-scanning mechanism or inducing complex instrument alignment. We demonstrate the MSM system with various phantom and biological specimens, and the results present effective resolution doubling, optical sectioning, and contrast enhancement. We anticipate MSM, as a highly accessible and compatible super-resolution technique, to offer a promising methodological pathway for broad cell biological discoveries.

## System Design and Methods

**Figure 1:**
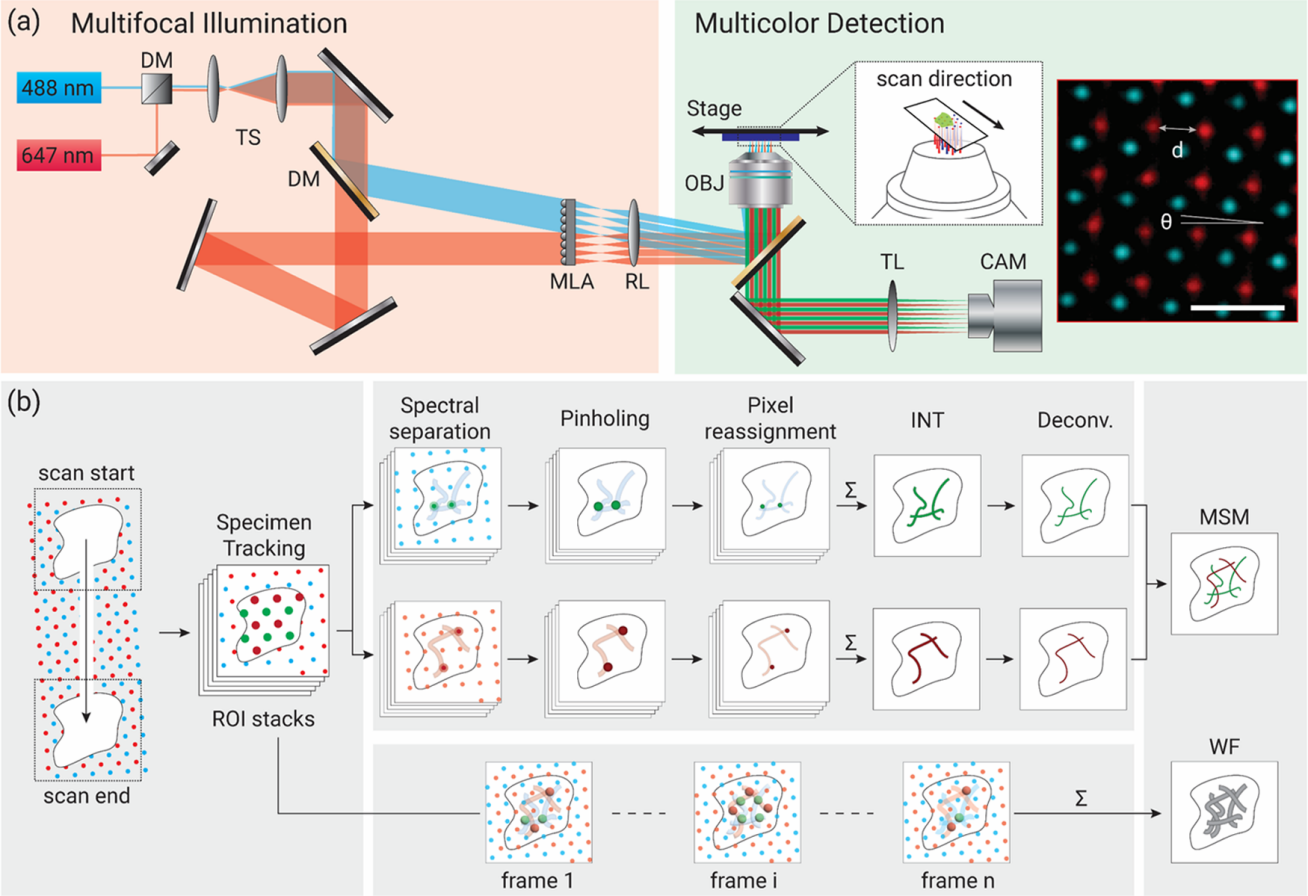
Multicolor multifocal scanning microscopy (MSM). (a) Optical setup of MSM. Multiple laser lines propagate and enter the microlens array (MLA) at different angles to form a multicolor foci excitation array at the sample plane (left inset). Right inset illustrates the experimental excitation pattern that contains an interposed two-color foci array (*d* = 1.6 μm and θ = 4°). DM, dichroic mirror; TS, telescope; RL, relay lens; OBJ, objective lens; TL, tube lens; and CAM, camera. (b) Data processing of MSM, containing image tracking, pinholing, pixel reassignment (scaling), summing to form the intermediate image (INT), and deconvolution. The overlay of tracked image stacks forms the wide-field (WF) image. Scale bar: 3 μm.

We constructed the MSM platform based on an epi-fluorescence microscope (Nikon Eclipse Ti2-U) that extends our recently proposed optofluidic scanning microscopy^20^ (Figures 1a and S1). In brief, the wide-field microscope was equipped with multicolor laser lines (488 and 647 nm, Coherent OBIS LX) and a 100×, 1.45NA objective lens (Nikon CFI Plan Apochromat Lambda 100× Oil). A microlens array (MLA, S100-f4-A, RPC Photonics) was placed in the illumination path of the setup, and the laser beams were relayed and propagated through the MLA at tilted angles with respect to the optical axis to form an interposed diffraction-limited multifocal multicolor excitation pattern (pitch *d* = 1.6 μm) at the sample plane (Figure 1a, inset). The fluorescent signals emitted from the sample were recorded by an sCMOS camera (Hamamatsu ORCA-Flash 4.0, effective sample pixel size = 6.5 μm/100 = 65 nm).

To acquire elemental multifocal images, the sample was placed on a high-precision motorized stage (ASI MS-2000-500) and scanned synchronously with the camera acquisition. Notably, the square patterns of each spectral foci were tilted by θ = 4° with respect to the scanning direction at a step interval of 0.1 μm and camera exposure time of 100 ms to facilitate seamless illumination coverage of the sample in both lateral dimensions^14,20^ (Figures 1a and S2). Notably, for MSM, to perform image reconstruction, unlike conventional spot-scanning ISM, the multifocal excitation patterns were first calibrated using a cover glass coated with uniformly distributed fluorescent dyes comprising multiple wavelengths. The acquired coordinates of the excitation foci were then recorded into separate spectral channels for processing multicolor samples.

The image processing of MSM contains four main procedures: image tracking, digital pinholing, pixel reassignment, and image deconvolution. Specifically, as illustrated in Figures 1b and S2, *first*, the field of view of interest is selected, and the motion of the elemental image is tracked based on the displacement of the stage per acquisition frame. The acquired images were processed to effectively form the spot-scanning condition for subsequent processing similar to conventional ISM. These images underwent flat-field correction according to a precalibrated illumination intensity envelope (Figure S3). *Second*, the tracked images undergo digital pinholes of 3 × 3 pixels, according to the precalibrated array of the excitation foci, to reject out-of-focus light. It should be noted that the corresponding wide-field images can be generated by merging the tracked images before the pinholing step (Figure 1b). *Third*, the pinholed images are locally contracted by a factor of two and reassigned to a scaled image of the halved pixel size of 32.5 nm (Figure S4).^14,20,21^*Lastly*, the resolution-enhanced (√2×) intermediate image can be produced by overlaying these pixel-reassigned images, which by a further step of blind deconvolution, can be processed to realize the full 2× resolution improvement over the diffraction limit of the corresponding wide-field image. Notably, the numerical PSF used in blind deconvolution is convenient for usage and offers an ideal SNR for reconstruction without minimal artifacts. Also, the interposed multicolor excitation pattern allows for the simultaneous acquisition of multiple spectral channels without ambiguity, which can be separately processed with the prior calibration and the above procedures. At last, these images are merged to form the final multicolor super-resolution image.

## Experimental Results

**Figure 2:**
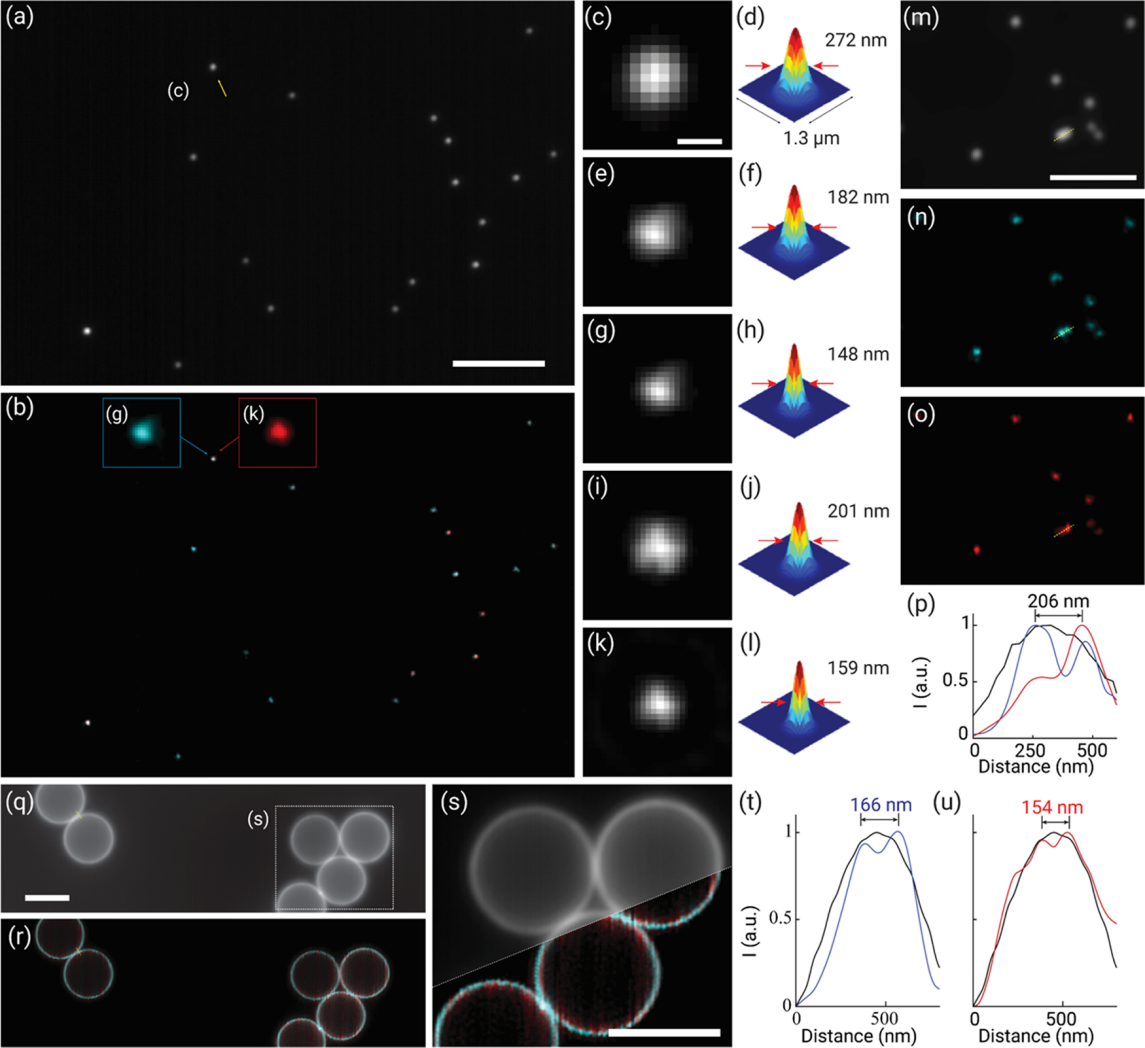
Multicolor imaging of phantom samples using MSM. (a,b) Wide-field (a) and MSM (b) images of 100 nm Tetraspek fluorescent beads with emission peaks at 515 nm (green) and 680 nm (dark red). (c–l) Zoomed-in wide-field (c), intermediate (e,i), and MSM (g,k) images of the bead as marked in panel (a,b), and on the right, their corresponding FWHMs by Gaussian fitting showing the enhanced resolution in both spectral channels. (m–p) Wide-field (m) and super-resolution (n,o) images exhibited two nearby beads separated at 206 nm below the diffraction limit that were resolvable by MSM (p). (q,r) Wide-field (q) and MSM (r) images of 6 μm surface-stained fluorescent microspheres. (s) Zoomed-in montage wide-field and super-resolution image of the boxed region as marked in panel (q), exhibiting enhanced resolution and contrast by MSM. (t,u) Cross-sectional intensity profiles along the yellow lines as marked in panels (q,r), showing resolved structures in both 515 nm (t) and 680 nm (u) channels. Scale bars: 5 μm (a), 300 nm (c), 3 μm (m), and 5 μm (q,s).

To characterize MSM, we first imaged 100 nm multispectral fluorescent beads (T7279, Thermo Fisher) and recorded the scanned elemental images under the multifocal excitation using 488 and 647 nm lasers. As seen, using MSM, the multicolor super-resolution images exhibited a higher contrast and improved resolution in both spectral channels, in comparison with the corresponding wide-field images (Figure 2a,b). In particular, the measurement displayed the full width at half-maximum (FWHM) values of the bead images taken by MSM at ∼140–160 nm in the blue and red channels, respectively, consistent with the predicted resolution doubling (∼130 nm) convolved with the 100 nm profile of the phantom structure. The results exhibited a nearly twofold improvement, as opposed to ∼272 nm for red color (286 nm, theoretically) as measured using the wide-field images (Figure 2c–l). In addition, nearby beads separated below the diffraction limit can be resolved in the multicolor MSM images (Figure 2m–p). Frequency analysis of the MSM images of these sub-diffraction-limited beads verified a consistent resolution doubling over their wide-field counterparts (Figure S5). Lastly, the MSM images of surface-stained 6 μm fluorescent microspheres (F24633, Thermo Fisher) showed the good alignment of the multicolor objects, as well as their enhanced optical sectioning and resolution of adjacent structures as close as 150–160 nm in both spectral channels (Figure 2q–u).

**Figure 3:**
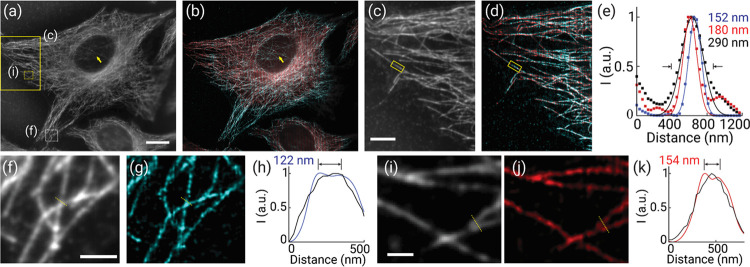
Super-resolution multicolor imaging of microtubules in HeLa cells using MSM. (a,b) Wide-field (a) and super-resolution (b) images of microtubules immune-stained for both 488 and 647 nm excitations. The arrows point to the thick nucleus region that exhibited enhanced image contrast and resolution of microtubules in the MSM image. (c,d) Zoomed-in wide-field (c) and super-resolution (d) images of the corresponding yellow boxed region as indicated in panel (a). (e) Cross-sectional intensity profiles of a microtubule filament in wide-field (black) and two-color super-resolution images. (f–k) Zoomed-in wide-field (f,i) and super-resolution (g,j) images of the corresponding boxed regions as marked in panel (a). Panels (h,k) show the cross-sectional intensity profiles along the corresponding dashed lines in panels (f,g) and (i,j), respectively. Scale bars: 10 μm (a), 5 μm (c), 2 μm (f), and 1 μm (i).

We next demonstrated multicolor imaging of biological samples with MSM. We first imaged microtubules in HeLa cells costained with both Alexa 488 and 647 (A32723 and A21235, Thermo Fisher, respectively). Compared with conventional wide-field images, super-resolution MSM imaging allows for the simultaneous multicolor acquisition of microtubules, and the reconstructed images demonstrated substantially enhanced contrast (e.g., the nucleus region) and subcellular resolution (Figures 3a,b and S6). As seen, the MSM images showed the colocalization of the delicate tubular structures revealed by both spectral labels (Figure 3c,d). Here, the colabeled individual filaments exhibited consistent sub-diffraction-limited FWHM values at 150–180 nm in both channels (Figure 3e), suggesting the agreement with the theoretical prediction of the immuno-stained microtubules (50–60 nm)^22^ convolved with the resolution (<150 nm) of the system. Furthermore, microtubule filaments that are separated as close as 122 and 154 nm can be well resolvable using MSM (Figure 3f–k), implying the twofold resolution enhancement over the diffraction limit, consistent with the measurements using phantom samples, as shown in Figure 2.

**Figure 4:**
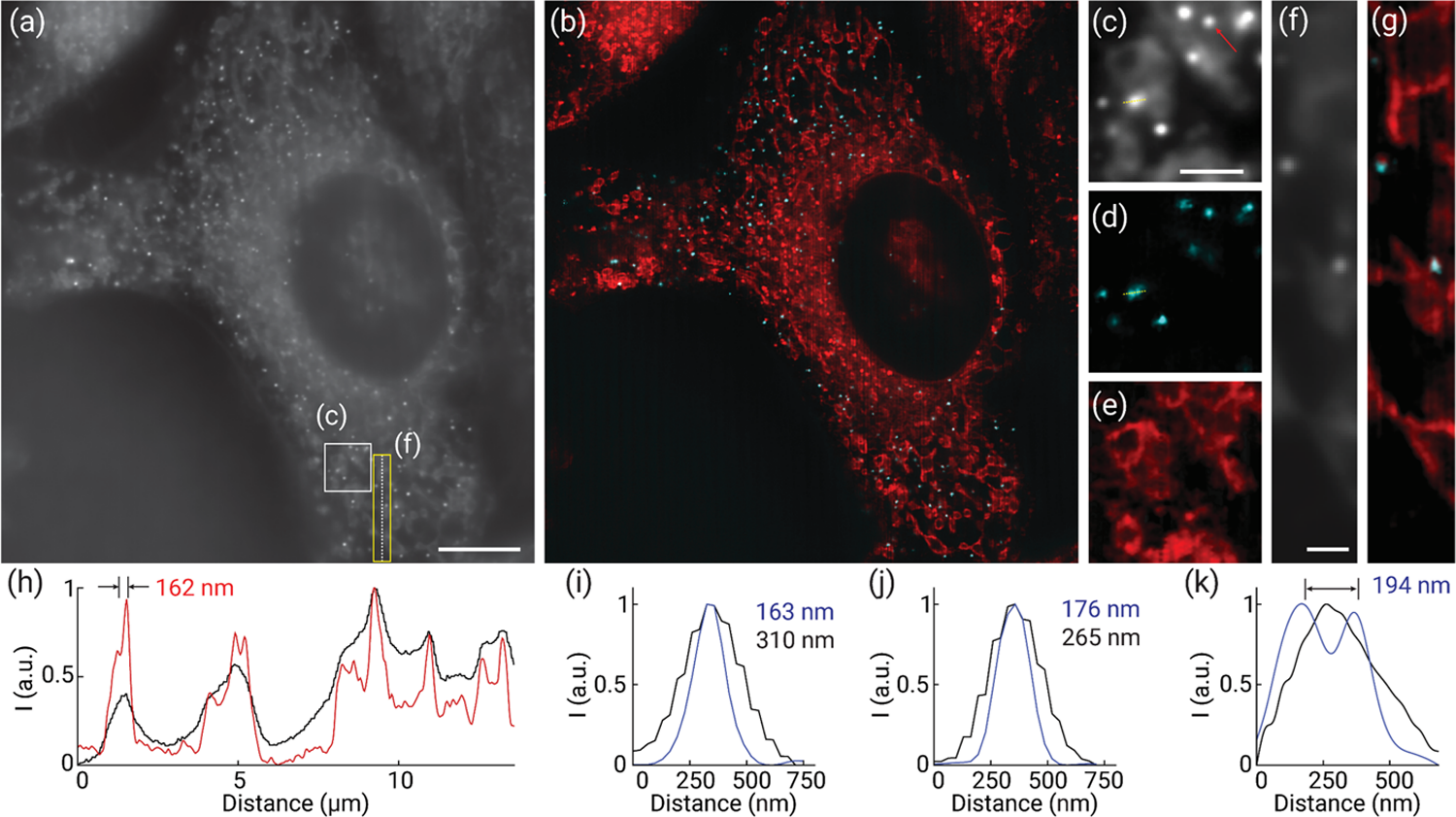
Super-resolution multicolor imaging of peroxisomes and mitochondria in HeLa cells using MSM. (a,b) Wide-field (a) and super-resolution (b) images of peroxisomes (green) and mitochondria (red) labeled with GFP and MitoTracker, respectively. (c–e) Zoomed-in wide-field (c) and super-resolution (d,e) images of the corresponding boxed region as indicated in panel (a). (f,g) Zoomed-in wide-field (f) and super-resolution (g) images of the corresponding yellow boxed region as indicated in panel (a), exhibiting enhanced optical sectioning and resolution. (h) Cross-sectional intensity profiles of mitochondria along the line as marked in panel (a), revealing fine structural details using MSM (red). (i,j) Transverse (*x* and *y*, respectively) cross-sectional intensity profiles of the peroxisome (indicated by the arrow in panel (c)) in both wide-field and super-resolution images. (k) Cross-sectional intensity profiles across the cluster of peroxisomes as indicated by the line in panels (c,d), showing the resolution of sub-diffraction-limited structures. Scale bars: 10 μm (a), 2 μm (c), and 1 μm (f).

Finally, we performed super-resolution MSM imaging of peroxisomes and mitochondria in HeLa cells. The interactions of the two organelles have recently been identified in various cell types to function closely in the regulation of cellular metabolism and signaling pathways.^23^ Here, MSM simultaneously acquired peroxisomes and mitochondria that were labeled with GFP (C10604, Thermo Fisher) and MitoTracker (M22426, Thermo Fisher), respectively (Figures 4 and S7). As seen, the two organelles were densely packed in the cellular space, becoming less distinguishable due to the low image contrast and resolution in the wide-field images (Figure 4a). On the contrary, the multifocal excitation and computational processing (pinholing and deconvolution) in MSM permitted effective image sectioning and enhanced resolution of the intracellular organelle details that are poorly detectable by wide-field microscopy (Figure 4a–g). For example, complex mitochondrial structural networks can be clearly displayed spanning the cells, and their finer features at 100–200 nm can be substantially recovered using MSM (Figure 4h). Meanwhile, the individual peroxisomes (typically 0.1–1 μm^24^) exhibited FWHM values at ∼170 nm, in comparison with the wide-field measurement at ∼300 nm (Figure 4i,j), and the clusters of closely located peroxisomes below the diffraction limit can be resolved using MSM (Figures 4k and S7). These results successfully demonstrate the ability of MSM to simultaneously visualize multiple subcellular structural details with substantially improved image quality and contents.

## Discussion and Conclusions

**Figure 5:**
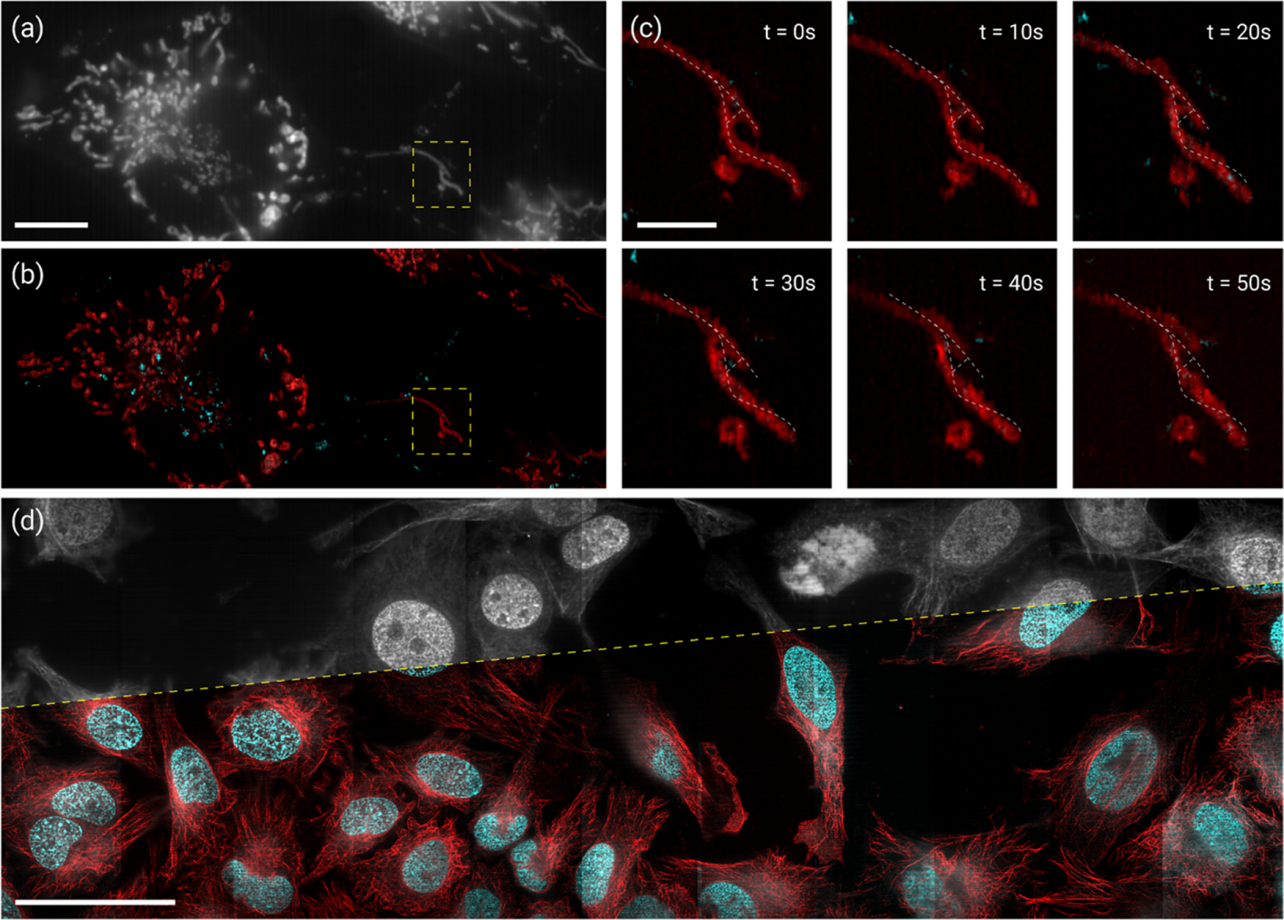
Multicolor MSM for live-cell imaging and with extended field of view. (a,b) Wide-field (a) and MSM (b) images of mitochondria (red) and lysosomes (green) of living HeLa cells. Recording images at a frame rate of 200 Hz, super-resolution sequences were formed every 1.3 s. (c) Zoomed-in time-lapse sequences of lysosomes and mitochondria in the boxed region in panel (c). The dashed lines marked the initial mitochondrial structure at *t* = 0, indicating the delicate motion of the organelle over time (Supplementary Videos S1 and S2). (d) Wide-field (top) and super-resolution (bottom) images of the nucleus (green) and microtubules (red) of HeLa cells across an extended field of view (>400 μm × 130 μm). Scale bars: 10 μm (a,b), 3 μm (c), and 50 μm (d).

In conclusion, we have developed MSM for simultaneous multicolor super-resolution imaging with effective resolution doubling, optical sectioning, and image contrast enhancement. As an advance of current ISM techniques, MSM presented a configuration accessible to commonly used epi-fluorescence microscopes and parallelized multicolor acquisition, avoiding complex implementation and compromise in imaging performance. The simultaneous multicolor scheme can be readily employed for live-cell imaging of multiple cellular organelles (Figure 5a–c). In addition, the sample translation configuration of MSM presents a unique strength in streamlining the fast acquisition and super-resolution imaging of a large field of view (Figure 5d). The method can be further integrated with extended spectral channels,^8^ barcode imaging,^25^ and optofluidics.^20^ The system is expected to achieve faster acquisition, higher spatiotemporal dimensions,^26^ efficient image processing and visualization, and the integration of versatile functionalities through improving the hardware and software design.^27−30^ We anticipate MSM to offer a promising methodological pathway for future super-resolution technology development and a broad range of cell biological discoveries.

## Supporting Information Available

Sample preparation protocols; list of optical components of the MSM system; details for data processing and code availability; additional resolution measurements; and live-cell image data sets (PDF)
SV1 (AVI)
SV2 (AVI)

## Supplementary Material

ph3c00205_si_001.pdf

ph3c00205_si_002.avi

ph3c00205_si_003.avi
