# Supplementary material for: Simultaneous Multicolor Multifocal Scanning Microscopy
Source: ACS Photonics. 2023 Jul 24;10(9):3035–41. doi: 10.1021/acsphotonics.3c00205 (PMC10515623; doi:10.1021/acsphotonics.3c00205)
Supplement: Supplementary file 1 — ph3c00205_si_001.pdf [file ph3c00205_si_001.pdf]

Supporting Information for:

Simultaneous Multicolor Multifocal Scanning Microscopy

Kyungduck Yoon[1,2,3], Keyi Han[1], Kidan Tadesse[1,2], Biagio Mandracchia[1], and Shu Jia[1,2,*]

1. Wallace H. Coulter Department of Biomedical Engineering, Georgia Institute of Technology and Emory University, Atlanta, Georgia, 30332

2. Parker H. Petit Institute for Bioengineering and Biosciences, Georgia Institute of Technology, Atlanta, Georgia, 30332

3. George W. Woodruff School of Mechanical Engineering, Georgia Institute of Technology, Atlanta, Georgia, 30332

E-mail: shu.jia@gatech.edu

Supplementary Note 1: Sample Preparation

Imaging of biological samples was performed with HeLa cells (Sigma-Aldrich, #93021013). The cells were cultured in 35 mm FluoroDish (World Precision Instruments, #FD35-100) in Dulbecco's modified Eagle medium (DMEM, Corning, #10-013-CV) with 10% fetal bovine serum (FBS, Corning, #35-011-CV) and 1% Penicillin-Streptomycin (Pen-Strep, ThermoFisher, #15140122) at 37 °C and in a 5% CO2 atmosphere. On the day of imaging two-color microtubules, the cells with first fixed with 0.3% (volume:volume) glutaraldehyde in extraction buffer, incubating for 1 minute at 37C. The extraction buffer consists 10 mM MES, 150 mM NaCl, 5 mM EDTA, 5 mM glucose, 5 mM MgCl2, and 0.25% (volume:volume) Triton X-100 in ultra-pure water. The buffer of the cells is switched to 2% (volume:volume) glutaraldehyde in cytoskeleton buffer at room temperature for 10 minutes. The cytoskeleton buffer is the aforementioned extraction buffer without the Triton X-100. The cells are then gently washed with blocking/permeability (b/p) solution for 5 minutes, 3 times. The b/p solution consists 2.5% (weight:volume) bovine serum albumin (BSA) and 0.1% (volume:volume) Triton X-100 in PBS. Then, the primary antibody that targets beta-tubulin was added to the cell dish with 2 mL of b/p solution at a concentration of 2 µg/mL (ThermoFisher, #32-2600). The cell dish was placed inside a humidified chamber at room temperature for 1 hour. After primary antibody tagging, the cell dish was washed with b/p solution 3 times, 5 minutes each time. Then the cells were labeled with 2 µg/mL of Goat anti-Mouse IgG conjugated with Alexa Fluor Plus 488 (ThermoFisher, #A32723) and 2 µg/mL of Goat anti-Mouse IgG conjugated with Alexa Fluor 647 (ThermoFisher, #A-21235) to achieve two-color staining. The staining took place inside the humidified chamber at room temperature for 1 hour. After secondary antibody labeling, the cell dish was washed with b/p solution three times 5 minutes each and sequentially washed with PBS twice. The sample is finally stored at 2 mL of PBS solution for imaging.

Peroxisome and mitochondria imaging was also performed with HeLa cells following the same cell culture protocol. Prior to the day of imaging, the cells were incubated in a pre-warmed (37 °C) mixed solution containing 3 mL modified DMEM and 20 µL CellLight Peroxisome-GFP (ThermoFisher, # C10604). The GFP was expressed on the peroxisomes in the cells after 18 hours of incubation. On the day of imaging, 0.6 µL of 1 mM MitoTracker Deep Red FM stains (ThermoFisher, #M22426) was added to the growth medium. The cells were incubated for additional 30 minutes. Then the growth medium was removed, and the cells were washed twice with clear Hank's balanced salt solution (HBSS, Corning, #21-021-CV). The cells were fixed 4% paraformaldehyde (PFA, diluted from 16% PFA with PFA:PBS:ultrapure-water in 1:2:1 ratio, Electron Microscopy Sciences) at room temperature for 12 minutes. The cells were washed twice with clear phosphate-buffered saline (PBS, Corning, #21-040-CM) and stored at 2.5 mL of PBS solution for imaging.

Fixed nucleus and microtubule imaging was performed with HeLa cells once they reached ~80% confluency. They were passaged and cultured in an 8-well glass-bottom μ-Slide (ibidi USA, #80827). When cells reached to ~60% confluency in the slide, they were washed with 500 μL culture medium once. Then, 250 nM of Syto 16 green stains (ThermoFisher, #S7578) in 200 μL culture medium was added to each well, incubating for 1 hour at 37 °C and in a 5% CO2 atmosphere. The cell fixation and immunostaining generally followed the N-STORM immunostaining protocol using the activator-reporter method. Briefly, each well was then washed with 500 μL PBS (Corning, #21-040-CV) once. Then, each well was fixed with 200 μL 3% PFA (Electron Microscopy Sciences):0.1% glutaraldehyde (Sigma-Aldrich, #G7651) in PBS at room temperature for 10 minutes. Extra aldehyde groups were reduced with 200 μL of 0.1% sodium borohydride (Sigma-Aldrich, #452882), followed by 3 times washing with PBS, 5 minutes each. After that, cells were blocked with blocking buffer (3% BSA (Sigma-Aldrich, #A7906) with 0.2% Triton X-100 (Fisher BioReagents, #BP151-100) in PBS) for 20 minutes. Then, 200 μL of primary antibody dilutions (BT7R, ThermoFisher, #MA5-16308, final concentration 10 μg/mL) in blocking buffer was added in each well and incubated for 30 minutes at room temperature, light avoided. Next, each well was washed 5 times with 200 μL washing buffer (0.2% BSA with 0.05% Triton X-100 in PBS) for 15 minutes per wash at room temperature. After washing, 150 μL of secondary antibody dilutions (ThermoFisher, #A-21236, final concentration 3 μg/mL) in blocking buffer was added in each well and incubated for 30 minutes at room temperature, light avoided. Then, each well was washed 3 times with 200 μL washing buffer for 10 minutes per wash at room temperature, followed by one time of washing in 500 μL PBS for 5 minutes. For better fluorescence imaging quality, cells were post-fix 200 μL 3% PFA:0.1% glutaraldehyde in PBS at room temperature for 10 minutes, followed by 3 times of washing in 500 μL PBS for 5 minutes per wash. Finally, cells were stored in 500 μL PBS for imaging purposes.

Live lysosomes with mitochondria and live actins with mitochondria imaging were also performed with HeLa cells following the same cell culture protocol. For the lysosomes with mitochondria, on the day of imaging, the imaging dish was first washed with a 2 mL culture medium once. Then, 50 nM MitoTracker Deep Red FM stains (ThermoFisher, #M22426) and 50 nM LysoTracker Green DND-26 (ThermoFisher, #L7526) in 2 mL culture medium was added to the imaging dish. The cells were incubated for 30 minutes at 37 °C and in a 5% CO2 atmosphere. Then, the culture medium was discarded, and the cells were washed twice with 2 mL FluoroBrite DMEM (ThermoFisher, #A1896701). Finally, 2 mL of FluoroBrite DMEM was added to the imaging dish for imaging purposes. In general, cells are good for imaging at room temperature for 1-2 hours.

On the day of imaging live actins and mitochondria, the imaging dish was first washed with a 2 mL culture medium once. Then, 50 nM MitoTracker Deep Red FM stains (ThermoFisher, #M22426) and 2 μL 1000X stock solution of CellMask Green Actin Tracking Stain (ThermoFisher, #A57243) in 2 mL culture medium were added to the imaging dish. The cells were incubated for 30 minutes at 37 °C and in a 5% CO_2 atmosphere. Then, the culture medium was discarded, and the cells were washed twice with 2 mL FluoroBrite DMEM (ThermoFisher, #A1896701). Finally, 2 mL of FluoroBrite DMEM was added to the imaging dish for imaging purposes. In general, cells are good for imaging at room temperature for ~1-2 hours.

Supplementary Note 2: List of MSM Components

Component	Manufacturer	Notes
**sCMOS camera**<br>Hamamatsu ORCA 4.0 V3	Hamamatsu	
**Lasers**<br>Coherent OBIS 1178769<br>Coherent OBIS 1185055	Coherent	
**Dichroic mirror 1**<br>T600lpxr-UF1	Chroma	Long pass filter cutoff frequency 600 nm<br>For the combination of excitation beams
**Dichroic mirror 2**<br>DMLP550L	Thorlabs	Long pass filter cutoff frequency 550 nm<br>For propagating at a non-orthogonal angle
**Dichroic mirror 3**<br>ZT 405/488/561/647 rpc-UF1<br>ZET 405/488/561/647 m	Chroma	For acquiring emission at detection
**Telescope lens 1**<br>AC254-030-A-ML	Thorlabs	Beam expander telescope 1
**Telescope lens 2**<br>AC254-500-A-ML	Thorlabs	Beam expander telescope 2
**Microlens array**<br>MLA-S100-f4-A-R1	RPC Photonics	
**Relay lens**<br>AC508-100-A-ML	Thorlabs	Paired with the microlens array
**Microscope**<br>Eclipse Ti2-U	Nikon	
**Objective lens**<br>CFI Plan Apochromat Lambda<br>100× Oil	Nikon	
**High-precision motorized stage**<br>MS-2000-500	ASI	

Supplementary Note 3: Details for Data Processing and Code Availability

As shown in **Figure S2**, during the acquisition, a stationary multifocal excitation of each wavelength tilted by 4° illuminates the sample, which is continuously translated by the motorized stage. As a result, roughly 260 images are required to scan the sample seamlessly. The spatially separated excitation foci of each wavelength successfully illuminate the entire field within the region of interest to reconstruct an image through pixel reassignment and further image processing procedures[1, 2]. The details of image processing for each individual color channel (shown in **Figure S4**) are listed as follows:

1. **Pinholing.** Before pixel reassignment, the region of interest is selected from the raw image that is synchronized with the displacement per frame generated by the motorized stage. Corresponding coordinates of the multifocal grids are used for rejecting out-of-focus light. A binary pinhole of 3 pixels × 3 pixels array, centered at the coordinate of each focus, is used for pinhole masking.
2. **Pixel Reassignment.** We digitally performed pixel reassignment to generate an intermediate image that exhibits √2× resolution improvement over the diffraction limit. The masked pixel values from the stack of the region of interest (e.g., $m \times n$) are padded into the new, half-pixel-sized (i.e., doubled pixel numbers $2m \times 2n$) image at the same locations.
3. **Spline interpolation.** The size of these pinhole masks is insufficient to generate a continuous, smooth intermediate image. The image may result in void pixels (black gaps), as shown in **Figure S4**, when for instance, imaging the phantom samples such as fluorescent microspheres. Note that the maximum gap of these bands does not exceed 3 pixels in the reassigned image (97.5 nm), which is well below the diffraction limit. These gaps are inpainted through a spline interpolation.
4. **Deconvolution.** The intermediate image is then processed with blind deconvolution to form the final super-resolution image with 2× resolution improvement over the diffraction limit.

For the reconstruction of MSM, we perform the pinhole masking, pixel reassignment, spline interpolation, and deconvolution for each wavelength and then merge the spectral channels to form the multicolor super-resolution image.

The software for MSM will be available at https://github.com/ShuJiaLab/MSM upon publication.

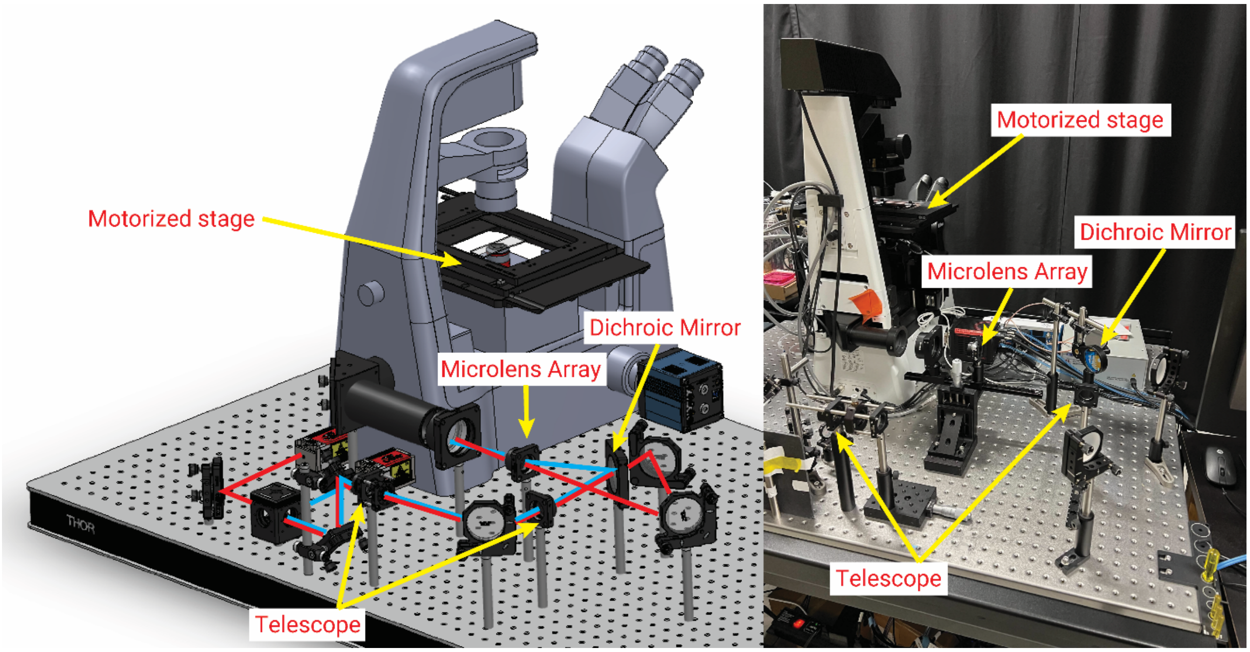

Figure S1: Design and photograph of the experimental setup. The collimated beams from the light sources are expanded by the telescope and reflected (or transmitted) by a dichroic mirror. The beams are independently controlled to form an interposed foci array at the sample plane. Multi-focal excitation at the sample plane remains stationary, and the sample scanning is performed using the motorized stage along one dimension.

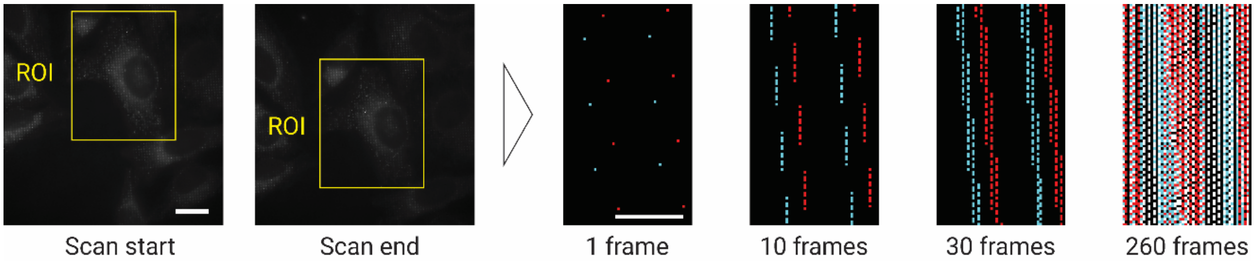

Figure S2: Detailed procedures of multicolor scanning in MSM. The sample is scanned by the motorized stage, which results in the continuous translation of the region of interest during the scanning procedure. Correspondingly, the coordinates of the multifocal excitation for each wavelength follows the motion of the region of interest every frame. Summing the trajectory of the multifocal excitation displays the coverage of the illumination on the sample plane. The sample plane is considered fully scanned, when the summed multifocal excitation covers the entire region of interest. Scale bars: 20 µm (left), 1.5 µm (right).

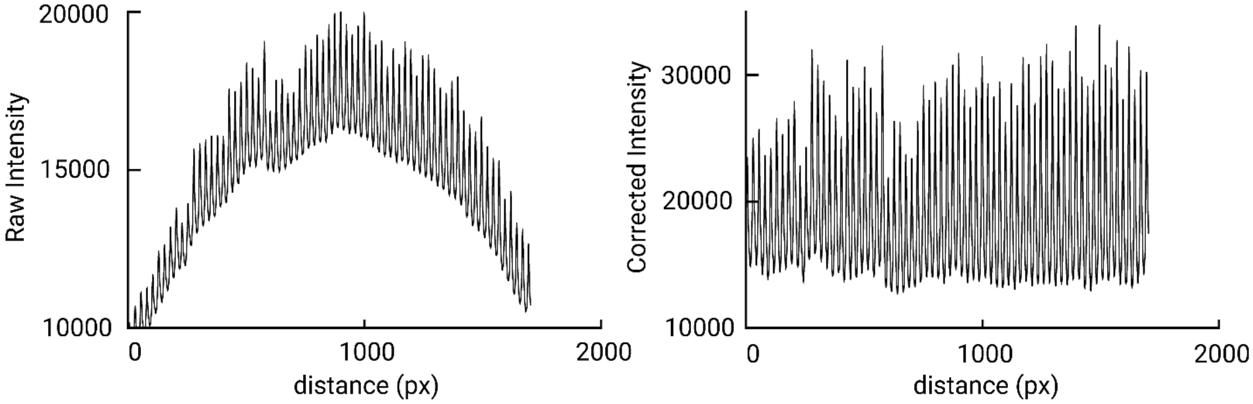

Figure S3: Intensity profile along a line of illumination foci (the peaks) before (left) and after (right) flat-field correction. The pre-calibrated intensity envelope is utilized for correcting raw images prior to pinholing.

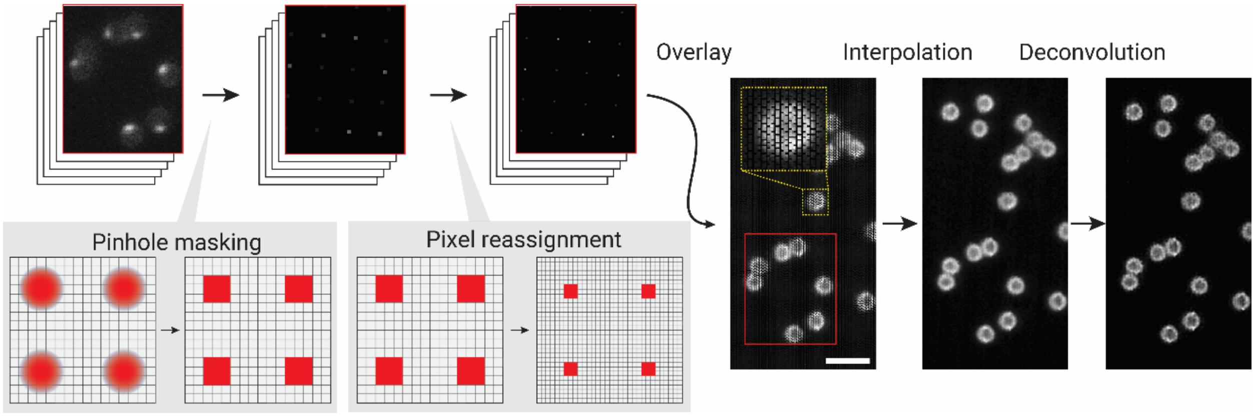

Figure S4: Details on image processing and reconstruction. A region of interest is selected from the raw image of a full-sized imaging sensor. For each frame, an array of 3×3 masks each centered at a focal location is applied to reject out-of-focus light and is further padded to same location on a new image of doubled pixel numbers for pixel reassignment. After overlaying the reassigned image sequence, void gaps are filled using spline interpolation to form the intermediate image with a resolution improvement of √2. This intermediate image is further deconvolved to form the super-resolution image. The example image was acquired using 1-μm fluorescent microspheres (F14791, ThermoFisher). Scale bar: 3 μm.

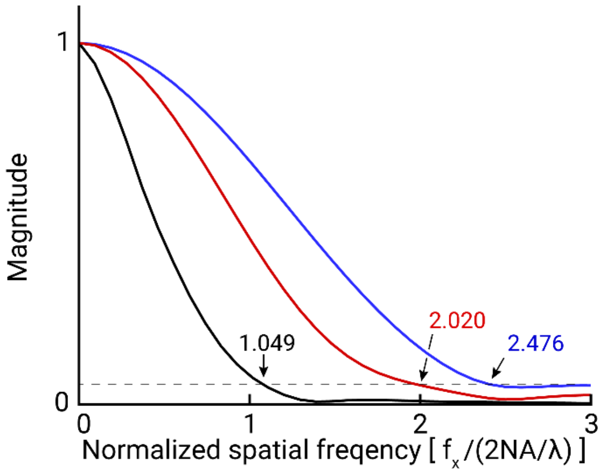

Figure S5: Frequency analysis of the MSM system. Spatial-frequency profiles are normalized by the theoretical diffraction-limited cutoff frequency of the 680-nm emission. Cutoff frequencies at 5% offset exhibit 1.049, 2.020, and 2.476 for the wide-field, red MSM, and blue MSM images, respectively.

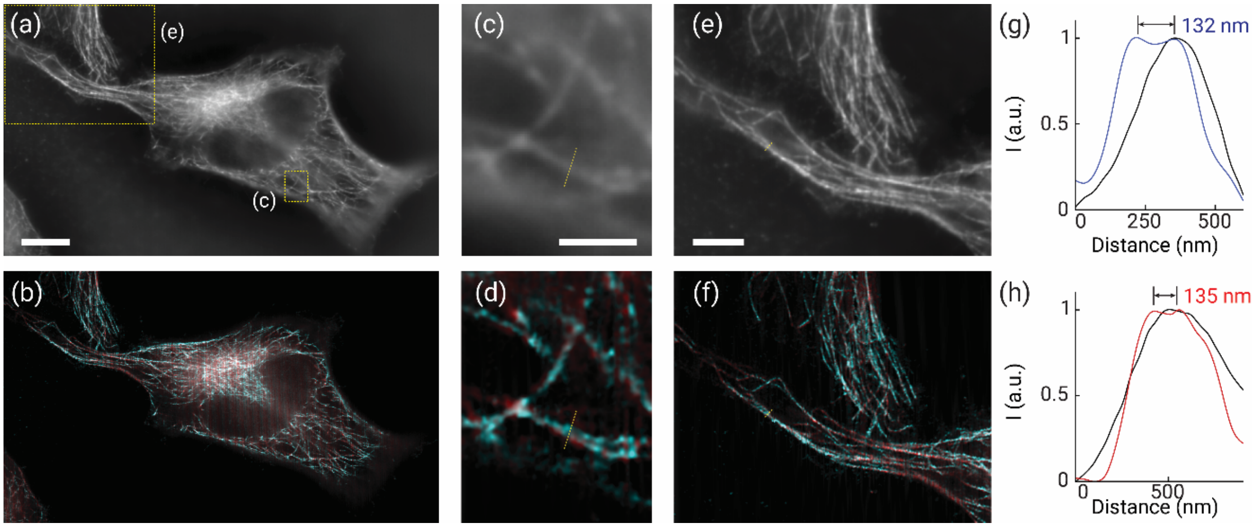

Figure S6: Super-resolution multicolor imaging of microtubules in HeLa cells using MSM. (a,b) Wide-field (a) and super-resolution (b) images of microtubules immuno-stained for both 488-nm and 647-nm excitations. (c-f) Zoomed-in wide-field (c,e) and super-resolution (d,f) images of the corresponding yellow boxed regions as indicated in (a). (g,h) show the cross-sectional intensity profiles along the corresponding dashed lines in (c,d) and (e,f), respectively. Scale bars: 10 µm (a), 2 µm (c), 5 µm (e).

126

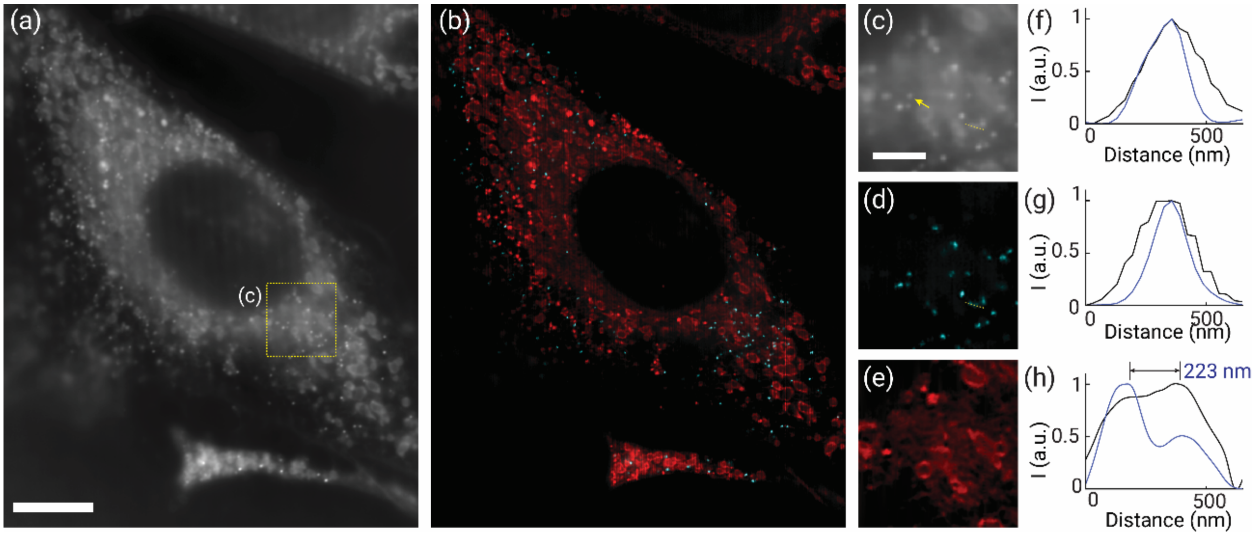

Figure S7: Super-resolution multicolor imaging of peroxisomes and mitochondria in HeLa cells using MSM. (a,b) Wide-field (a) and super-resolution (b) images of mitochondria (red) and peroxisomes (green) labelled with MitoTracker and GFP, respectively. (c-e) Zoomed-in wide-field (c) and super-resolution (d,e) images of the corresponding boxed region as indicated in (a). Vertical (FWHMs: 201 nm (MSM), 274 nm (wide-field)) and horizontal (FWHMs: 169 nm (MSM), 273 nm (wide-field)) intensity profiles of a single peroxisome arrow-marked in (c) are plotted in (f-g), and the separation of adjacent peroxisomes below the diffraction limit in (c-d) are shown in (h). Scale bars: 10 µm (a), 3 µm (c).

Supplementary Video S1: Time-lapse recording of lysosomes (blue) and mitochondria (red) in live HeLa cells, acquired at 200 fps for 50 sec.

Supplementary Video S2: Time-lapse recording of actin filaments (blue) and mitochondria (red) in live HeLa cells, acquired at 200 fps for 90 sec.

Supplementary References:

1. Mandracchia, B., Son, J. & Jia, S. Super-resolution optofluidic scanning microscopy. *Lab Chip* **21**, 489-493 (2021).
2. York, A.G. et al. Resolution doubling in live, multicellular organisms via multifocal structured illumination microscopy. *Nat Methods* **9**, 749-754 (2012).
